# Shape-shifting corals: Molecular markers show morphology is evolutionarily plastic in *Porites*

**DOI:** 10.1186/1471-2148-9-45

**Published:** 2009-02-24

**Authors:** Zac H Forsman, Daniel J Barshis, Cynthia L Hunter, Robert J Toonen

**Affiliations:** 1 Hawaii Institute of Marine Biology, PO Box 1346, Kaneohe, HI 96744, USA; 2 Biology Program, University of Hawaii at Manoa, Honolulu, HI 96822, USA; 3 Zoology Department, University of Hawaii at Manoa, Honolulu, HI 96822, USA

## Abstract

**Background:**

Corals are notoriously difficult to identify at the species-level due to few diagnostic characters and variable skeletal morphology. This 'coral species problem' is an impediment to understanding the evolution and biodiversity of this important and threatened group of organisms. We examined the evolution of the nuclear ribosomal internal transcribed spacer (ITS) and mitochondrial markers (COI, putative control region) in *Porites*, one of the most taxonomically challenging and ecologically important genera of reef-building corals.

**Results:**

Nuclear and mitochondrial markers were congruent, clearly resolving many traditionally recognized species; however, branching and mounding varieties were genetically indistinguishable within at least two clades, and specimens matching the description of '*Porites lutea*' sorted into three genetically divergent groups. Corallite-level features were generally concordant with genetic groups, although hyper-variability in one group (Clade I) overlapped and obscured several others, and *Synarea *(previously thought to be a separate subgenus) was closely related to congeners despite its unique morphology. Scanning electron microscopy revealed subtle differences between genetic groups that may have been overlooked previously as taxonomic characters.

**Conclusion:**

This study demonstrates that the coral skeleton can be remarkably evolutionarily plastic, which may explain some taxonomic difficulties, and obscure underlying patterns of endemism and diversity.

## Background

Coral species are notoriously difficult to identify, which is an impediment to understanding their ecology, evolution, and biodiversity. Coral species descriptions are traditionally based solely on skeletal morphology, which is known to exhibit variation unrelated to reproductive isolation or evolutionary divergence [1-3]. Coral skeletal morphology can be remarkably phenotypically plastic, responding to a wide variety of parameters such as light, sedimentation, water motion, water chemistry, and ecological interactions [2,4]. Adding to taxonomic confusion, the conceptual nature of the coral species is a subject of intense debate, particularly regarding the permeability of reproductive boundaries between morphological 'species'. Molecular tools are clearly needed to delineate species boundaries, and to reveal patterns of their evolution and biodiversity. However, molecular studies have met with considerable technical challenges, and fueled additional controversy over the extent and evolutionary significance of hybridization between species [4-6].

Mitochondrial DNA is one the most widely used and informative molecular markers in Metazoa; however, the Anthozoan mitochondrial genome evolves unusually slowly, providing little phylogenetic resolution at or below the family or genus level [7,8]. The nuclear ribosomal ITS region (a portion of the ribosomal cistron consisting of two rapidly evolving internal transcribed spacers and the 5.8S gene) is among the most widely used molecular markers for species-level studies in plants [9], fungi [10], and corals [11-29]. Despite wide use of the ITS region, the multi-copy marker presents challenges, particularly when disparate variants are found within a single genome. Highly divergent ITS copies are thought to be found exclusively within organisms that have a history of hybridization between disparate parents [30,31], and the thousands of copies in a typical Eukaryotic genome are thought to be homogenized by recombinant processes (concerted evolution) in frequently interbreeding groups [32,33].

Alvarez & Wendel [9] and Vollmer & Palumbi [34] cautioned however that it is difficult to distinguish between introgression from hybridization and incomplete lineage sorting of ancestral polymorphisms. Vollmer & Palumbi [34] argued against using the ITS region in corals altogether based on observations in *Acropora*, a genus with many species that are known to hybridize [6,35]. However, *Acropora *contains the highest known levels of ITS intra-genomic variation observed in Metazoa [13] from pseudogenes originating from an ancient hybridization event [24]. A broad survey of ITS intra-genomic variation in corals showed that problematic variation was a rare exception found only in *Acropora *and not as a general rule in other coral taxa [29]. ITS intra-genomic variation is low in *Porites *and the phylogenetic signal is strong relative to noise from alignment ambiguities, which may be reduced with increased taxonomic sampling [21,23].

The majority of species-level phylogenetic studies on corals failed to resolve closely related morphospecies, which has fueled debate about the nature of the coral species, and raised questions about the evolution of the most widely used molecular markers. The present study examines congeneric species across a range of evolutionary divergences with comparatively broad taxonomic sampling to determine if skeletal morphology is evolutionarily constrained or labile. We explore patterns of evolution and biodiversity in *Porites *with both the ITS region and mitochondrial markers (cytochrome c oxidase subunit I and the 'putative control region').

*Porites *provides an excellent example of the 'species problem' in corals [36], where highly variable morphology defies classification into discrete species groups. *Porites *provide a major structural component of coral reefs worldwide and are among the most ecologically important (in terms of abundance, global distribution, and variety of habitats occupied) yet taxonomically challenging genera of reef building coral.

## Results

Three hundred and seven ITS region sequences from 91 individual coral colonies were aligned and compared (Table 1). Redundant (99% identical or higher) ITS region sequences within each individual were omitted from the alignments, leaving 188 sequences for phylogenetic analysis. The non-gap coded ITS alignment was 895 sites long, with 440 variable and 317 parsimony informative sites. Coding gaps resulted in no major changes in topology, but resulted in slightly higher clade support values for some deeper nodes (not shown). Pair-wise nucleotide distance between sequences within individual colonies was uniformly low (mean intra-genomic distance = 0.005 ± 0.001 SE). Pair-wise nucleotide distance between individuals within significant clades (mean intra-specific distance = 0.009 ± 0.002 SE), was an order of magnitude lower than the distance between significant clades (mean inter-specific distance = 0.118 ± 0.005 SE).

**Table 1:** Table of samples collected and GenBank Accession numbers

						**Genbank Accession Number**		
**Code**	**Identification**	**Location**	**Collector**	**C**	**D**	**ITS**	**mtCOI**	**mtCR**
a1	*p. lobata*	Australia	M. Takabayashi	4		AY320310–AY320312		
a2	*P. lobata*	Australia	M. Takabayashi	3		AY320306–AY320309		
acl	*P. lutea*	Taiwan	A. Chen, J. Veron	1		FJ416498		
as17	*P. lobata*	American Samoa	D. Barshis	10		FJ416499–FJ416502		
as18	*P. solida*	American Samoa	D. Barshis	3		FJ416503–FJ416504	FJ423962	FJ427364
as23	*P. sp2*	American Samoa	C. Birkeland	1		FJ416505		
as29	*P. lichen*	American Samoa	C. Birkeland	2		FJ416506–FJ416507	FJ423963	FJ427365
as30	*P. lichen*	American Samoa	C. Birkeland	5		FJ416508–FJ416512	FJ423987	FJ427389
as31	*P. annae*	American Samoa	C. Birkeland	3		FJ416513–FJ416515	FJ423964	FJ427366
as32	*P. annae*	American Samoa	C. Birkeland	3		FJ416516–FJ416518	FJ423965	FJ427367
as35	*P. sp2*	American Samoa	C. Birkeland	4		FJ416519–FJ416522	FJ423966	FJ427368
as36	*P. lutea*	American Samoa	C. Birkeland	4		FJ416523–FJ416526	FJ423967	FJ427369
as43	*P. cylindrica*	American Samoa	T. Oliver	5		FJ416527	FJ423968	FJ427370
asb1	*P. sp2*	American Samoa	D. Barshis	2		FJ416528		
asb11	*P. sp2*	American Samoa	D. Barshis	10		FJ416529–FJ416532		
asb12	*P. lutea*	American Samoa	D. Barshis	2		FJ416533–FJ416534		
asb13	*P. lutea*	American Samoa	D. Barshis	3		FJ416535–FJ416537		
asb14	*P. solida*	American Samoa	D. Barshis	11		FJ416538		
asb15	*P. solida*	American Samoa	D. Barshis	8		FJ416539		
asb16	*P. solida*	American Samoa	D. Barshis	4		FJ416540		
asb17	*P. solida*	American Samoa	D. Barshis	12		FJ416541		
asb3	*P. lutea*	American Samoa	D. Barshis	10		FJ416542–FJ416543		
asb4	*P. lutea*	American Samoa	D. Barshis	7		FJ416544–FJ416545		
asb5	*P. lutea*	American Samoa	D. Barshis	9		FJ416546–FJ416547		
asb6	*P. lutea*	American Samoa	D. Barshis	7		FJ416548–FJ416549		
asb7	*P. lutea*	American Samoa	D. Barshis	8		FJ416550–FJ416552		
asb8	*P. lutea*	American Samoa	D. Barshis	8		FJ416553–FJ416554		
asb9	*P. cylindrica*	American Samoa	D. Barshis	10		FJ416555–FJ416556		
b6	*P. astreoides*	Belize	G. Wellington	3		AY458024–AY458026		
bj2	*P. sverdrupi*	Baja California	B. Victor	2		AY458047–AY458048		
bj4	*P. sverdrupi*	Baja California	B. Victor	2		AY458049–AY458050		
bj7	*P. sverdrupi*	Baja California	B. Victor	3		AY458051–AY458053		
br1	*P. astreoides*	Brazil	E. Neves	3		AY458027–AY458029		
br4	*P. astreoides*	Brazil	E. Neves, R. Johnsson	2		AY458030–AY458031		
br6	*P. astreoides*	Brazil	E. Neves	2	1	AY458035–AY458036	FJ423961	FJ427363
cit	*P. compressa*	Hawaii, Oahu	Z. Forsman, C. Hunter		1	FJ416557	FJ423970	FJ427372
colP1	*P. colonensis*	Panama, Caribbean	J. Mate, H. Guzman	2		AY458060–AY458061		
colP3	*P. colonensis*	Panama, Caribbean	J. Mate, H. Guzman	2		AY458062–AY458063	FJ423972	FJ427374
dk2	*P. duerdeni*	Hawaii, Oahu	C. Hunter, J. Maragos		1	FJ416558		
e121	*P. lobata*	Easter Island	G. Wellington	1		AY320305		
e20	*P. lobata*	Easter Island	G. Wellington	3		AY320296–AY320298		
e47	*P. lobata*	Easter Island	G. Wellington	3		AY320299–AY320301	FJ423973	FJ427375
e48	*P. lobata*	Easter Island	G. Wellington	3		AY320302–AY320304	FJ423974	FJ427376
fg1	*P. astreoides*	Gulf of Mexico	T. Shearer	3	1	AY458021–AY458023		
fj4	*P. lobata*	Fiji	G. Wellington	3		AY320346–AY320348		
fj6	*P. lobata*	Fiji	G. Wellington	2		AY320349–AY320350		
fj7	*P. lobata*	Fiji	G. Wellington	2		AY320351–AY320352		
g3	*P. lobata*	Galapagos	Z. Forsman, G. Wellington	5		AY320331–AY320336		
g66	*P. lobata*	Galapagos	Z. Forsman, G. Wellington	3		AY320337–AY320339		
g7	*P. lobata*	Galapagos	Z. Forsman, G. Wellington	3		AY320340–AY320342		
g8	*P. lobata*	Galapagos	Z. Forsman, G. Wellington	3		AY320343–AY320345		
hm100	*P. evermanni*	Hawaii, Maui	Z. Forsman, D. Fenner	5		FJ416559–FJ416563		
hm99	*P. lobata*	Hawaii, Maui	Z. Forsman, D. Fenner	2		FJ416579		
hm19	*P. annae*	Hawaii, Maui	Z. Forsman, D. Fenner	3	1	FJ416564–FJ416565	FJ423975	FJ427377
hm20	*P. hawaiiensis*	Hawaii, Maui	Z. Forsman, D. Fenner	6		FJ416566–FJ416571	FJ423981	FJ427383
hm28	*P. duerdeni*	Hawaii, Maui	Z. Forsman, D. Fenner		1	FJ416572	FJ423976	FJ427378
hm29	*P. duerdeni*	Hawaii, Maui	Z. Forsman, D. Fenner		1	FJ416573	FJ423977	FJ427379
hm35	*P. solida*	Hawaii, Maui	Z. Forsman, D. Fenner		1	FJ416574	FJ423978	FJ427380
hm54	*P. hawaiiensis*	Hawaii, Maui	Z. Forsman, D. Fenner	1		FJ416575	FJ423979	FJ427381
hm55	*P. rus*	Hawaii, Maui	P. Reath, D. Fenner		1	FJ416576	FJ423980	FJ427382
hm56	*P. brighami*	Hawaii, Maui	P. Reath, D. Fenner		1	FJ416577		
hm6	*P. monticulosa*	Hawaii, Maui	P. Reath, D. Fenner		1	FJ416578		
l13	*P. compressa*	Hawaii, Oahu	Z. Forsman		1	FJ416580		
l2	*P. compressa*	Hawaii, Oahu	Z. Forsman		1	FJ416581	FJ423971	FJ427373
l3	*P. compressa*	Hawaii, Oahu	Z. Forsman	3	1	FJ416582	FJ423982	FJ427384
l4	*P. lobata*	Hawaii, Oahu	Z. Forsman	3	1	FJ416583–FJ416585	FJ423983	FJ427385
l5	*P. evermanni*	Hawaii, Oahu	Z. Forsman		1	FJ416586	FJ423984	FJ427386
l6	*P. evermanni*	Hawaii, Oahu	Z. Forsman	4	1	FJ416587–FJ416590	FJ423985	FJ427387
l7	*P. annae*	Hawaii, Oahu	Z. Forsman		1	FJ416591	FJ423986	FJ427388
l9	*P. compressa*	Hawaii, Oahu	Z. Forsman		1	FJ416592		
p1	*P. furcata*	Panama, Caribbean	C. Guevara	3		AY458044–AY458046	FJ423988	FJ427390
p10	*P. astreoides*	Panama, Caribbean	C. Guevara	2		AY458033–AY458034		
p2	*P. astreoides*	Panama, Caribbean	C. Guevara	1		AY458032	FJ423989	FJ427391
pan75	*P. panamensis*	Panama, Pacific	J. Mate, H. Guzman	3		AY458054–AY458056	FJ423990	FJ427392
pb4	*P. divaricata*	Belize	G. Wellington	3		AY458041–AY458043	FJ423969	FJ427371
pb7	*P. divaricata*	Belize	G. Wellington	3		AY458037–AY458039		
pb9	*P. divaricata*	Belize	G. Wellington	1		AY458040		
pp18	*p. sp*	Panama, Pacific	G. Wellington	2		AY320289–AY320291		
pp19	*p. sp*	Panama, Pacific	G. Wellington	3	1	AY320291–AY320293	FJ423991	FJ427393
pp47	*p. sp*	Panama, Pacific	G. Wellington	1		AY320295	FJ423992	FJ427394
pp48	*p. sp*	Panama, Pacific	G. Wellington	1		AY320294		
r1	*P. lobata*	Rarotonga	G. Wellington	3		AY320313–AY320315		
r4	*P. lobata*	Rarotonga	G. Wellington	3		AY320316–AY320318		
r6	*P. lobata*	Rarotonga	G. Wellington	3		AY320319–AY320321		
rus1	*P. rus*	Tahiti	B. Victor	2		AY458057–AY458058	FJ423993	FJ427395
rus2	*P. rus*	Tahiti	B. Victor	1		AY458059		
t2	*P. lobata*	Tahiti	G. Wellington	3		AY320322–AY320324	FJ423994	FJ427396
t3	*P. lobata*	Tahiti	G. Wellington	3		AY320325–AY320327		
t6	*P. lobata*	Tahiti	G. Wellington	3		AY320328–AY320330		
wa3	*Goniopora spp*.	Fiji	C. Delbeek	1		FJ416593	FJ423995	FJ427397
wa4	*P. cylindrica*	Fiji	C. Delbeek		1	FJ416594	FJ423996	FJ427398

C = number of molecular clones, D = direct sequenced.

**Figure 1:**
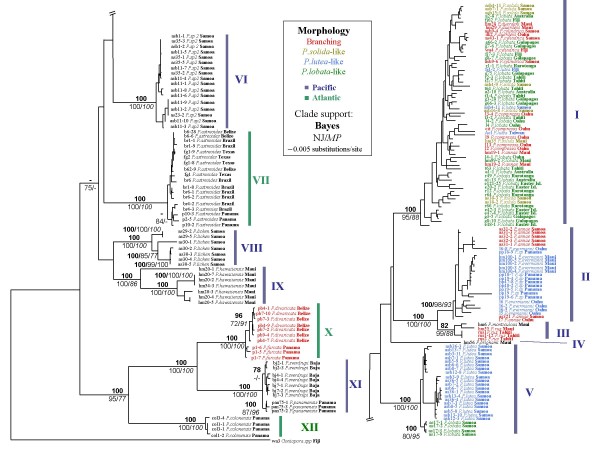
**Bayesian phylogenetic tree of the ITS data set**. The sample identification code is followed by a dash and a clone number for cloned sequences; redundant sequences within an individual have been removed. Blue lines represent Pacific taxa and green lines represent Atlantic taxa. Colonies with branching morphology are highlighted in red. Specimens that were identified as *P. solida*, *P. lutea *and *P. lobata *are highlighted in light green, blue, and green respectively. Numbers above each node represent Bayesian posterior probability values; below left are NJ bootstrap values, below right are MP bootstrap values. Clade support values below 70, or those that do not include all molecular clones from an individual, are not shown.

**Figure 2:**
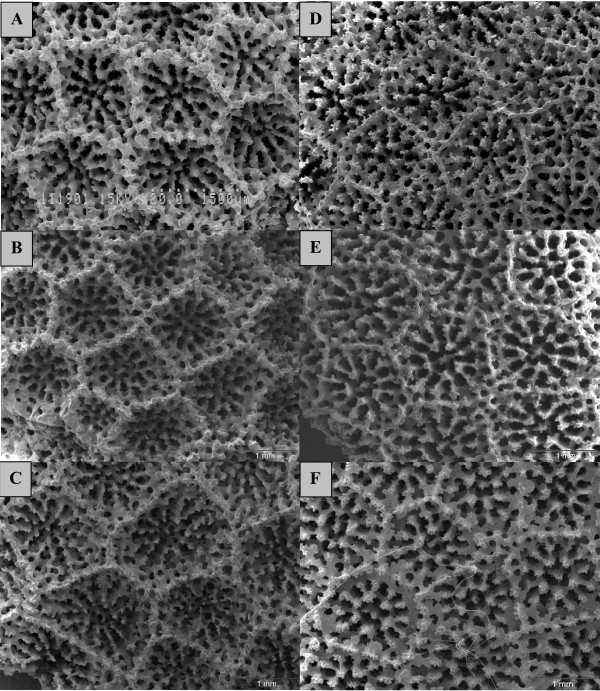
**SEM images of *Porites *corallites**. A. *P. compressa *(Hawaii); B. *P. solida *(Hawaii); C. *P. lobata *(Hawaii, syntype Bishop Museum SC454); D. *P. lutea *(Marshall islands, Bishop Museum SC1329); E. *P. sp *(Panama); F. *P. evermanni *(Hawaii).

ITS phylograms supported traditional species designations, with the exception of four Pacific clades: I, II, III and V (Figure 1). Branching and mounding morphospecies were genetically indistinguishable within these clades, yet deep genetic differences were observed in corals with very similar colony morphology and corallite appearance. Clade I contained both branching and mounding morphospecies collected across a broad geographic range (Caribbean, Eastern Pacific, Hawaii, Indo-West Pacific). Branching morphospecies (*P. cylindrica*, *P. compressa*, *P. duerdeni*, and *P. annae*) were genetically indistinguishable from each other, and from mounding morphospecies (*P. lobata*, and *P. solida*). *P. compressa *and *P. duerdeni *are thought to be endemic to Hawaii [37], yet they exhibited no fixed differences from other samples in Clade I. Both colony and corallite-level characters appeared highly variable within this clade. Corallite-level characters such as number or size of pali, free or fused triplets (see [4] for an illustration of taxonomic characters) were highly variable, often within the same colony, although all Clade I specimens tended to have relatively high corallite walls (Figure 2). Clade I exhibited morphological variation that often overlapped and obscured Clades II, and V. Clade I corallite appearance ranged from *P. solida-like *(large deep calyx, short pali, free triplet) to *P. lutea-like *(shallow calyx, tall pali, fused triplet) at opposite ends of a spectrum with *P. lobata-like *morphology as an intermediate.

Clade II also contained a mixture of branching and mounding corals (mounding *P. evermanni *from Hawaii, *P. sp *from Panama, and branching *P. annae *from Samoa and Hawaii). *P. evermanni *is either thought to be a Hawaiian endemic [4], or a synonym of *P. lutea *[37]. *P. evermanni *was genetically indistinguishable and morphologically very similar to *P. sp *from Panama, and genetically indistinguishable from the branching Samoan *P. annae*, yet it was deeply divergent from *P. lutea *from Samoa (Clade V). Traditional corallite-level characters in Clade II were similar to some individuals in the highly variable Clade I; however, scanning electron microscopy revealed subtle differences between Clade II and other clades; walls, denticles, pali, and columella tended to have similar height, with more evenly spaced and deeper interstitial holes (Figure 2).

Clade III contained *P. monticulosa *and *P. rus*, which are so distinct at the corallite level that they have been considered to belong to a separate subgenus (*Synarea*). Clade III was, unexpectedly, a closely related sister group to Clade II. Clade IV contained *P. brighami *(only one individual), a species that forms small (< 10 centimeters diameter) gray colonies with deep funnel-like calices. Clade V consisted primarily of *P. lutea *from Samoa (including an extremely large colony, approximately 19 m in diameter) and an individual identified as *P. lobata *that was genetically distinct from other Clade V individuals. Clade VI consisted of *P. sp2*, a putative new species that forms small colonies with stout small branches and shallow calices. Clade VII contained two sub-clades of *P. astreoides*; one contained individuals from Belize, the Gulf of Mexico, and Brazil, while the other contained individuals from Brazil and Panama. Clade VIII contained *P. lichen *from Samoa, with two divergent ITS sequence types with one intermediate (possibly recombinant) sequence. Clade IX consisted of *P. hawaiiensis *(which is referred to as *P.cf.bernardi *by Fenner [37]), a brooding species with crawling larvae (pers. obs.) that forms very small (< 10 centimeters in diameter) encrusting colonies. Clade X contained a monophyletic group of *P. divaricata *from Belize, nested within *P. furcata *from Panama (NJ trees, not shown, show weakly supported reciprocally monophyletic groups). Clade XI contained reciprocally monophyletic groups of *P. sverdrupi *from Mexico, and *P. panamensis *from the Pacific coast of Panama. Clade XII contained only *P. colonensis *from the Atlantic coast of Panama.

**Figure 3:**
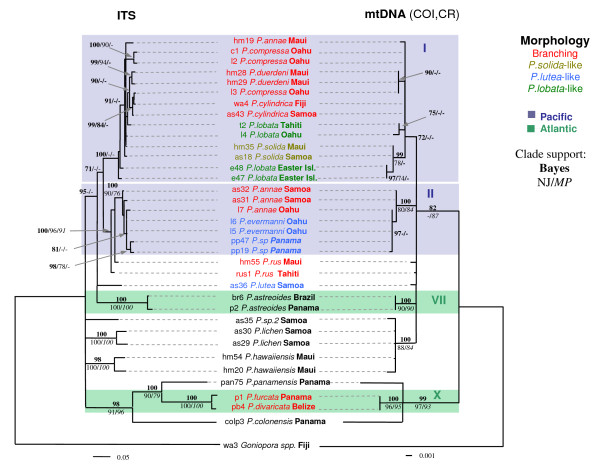
**Bayesian phylogenetic tree of ITS and mitochondrial sequences**. Blue lines represent Pacific taxa and green lines represent Atlantic taxa. Colonies with branching morphology are highlighted in red. Specimens that match the species descriptions of *P. solida*, *P. lutea *and *P. lobata *are highlighted in light green, blue, and green respectively. Numbers above each node represent Baysian posterior probability values; below left are NJ bootstrap values, below right are MP bootstrap values. Clade support values below 70 are not shown.

Nuclear and mitochondrial data sets had a very similar overall topology (Figure 3). The COI alignment consisted of 638 sites, 38 of which were variable, and 22 were parsimony informative. The 'putative control region' alignment had 293 sites, 41 of which were variable, and 13 were parsimony informative. A partition homogeneity test indicated no significant differences between the mitochondrial and ITS data sets (missing and ambiguous characters excluded, *p *= 0.349; included, *p *= 0.298). The ITS tree had longer branch lengths, more significant groups, and fewer polytomies than the mitochondrial tree. The mitochondrial data supported many of the same clades as the ITS data sets, although there was some discordance between nuclear and mitochondrial trees, particularly within Clade I. Clade I and II in the mitochondrial tree were more consistent with divisions between branching and mounding morphologies, although not reciprocally monophyletic and not supported by NJ and MP trees (Figure 3). The mitochondrial data were unable to resolve *P. sp.2*, *P. lichen*, and *P. hawaiiensis*, whereas the ITS data showed deep divergence between the three taxa.

## Discussion

In contrast to most molecular studies on corals, the present study finds clear genetic breaks and deep divergence between many congeneric species, which is likely to have resulted from taxonomic sampling across a comparatively broad range of evolutionary divergence, and geographic regions. The majority of genetic breaks are consistent with morphospecies designations, with the exception of three Pacific clades (Clade I, II and V) that exhibit highly variable colony morphology. Several shallow Pacific clades contained both branching and mounding varieties that were genetically indistinguishable. Conversely, specimens that appeared very similar (matching the species description of *P. lutea*) sorted into several clades that were deeply genetically divergent.

Corallite-level traits appeared more broadly consistent with genetic groups although these traits also exhibited rapid evolution and high variability, particularly within Clade I and between Clade I, II, and III. *P. monticulosa *and *P. rus *are so distinct from other *Porites *at the corallite-level, that Verrill [38] placed them into a separate subgenus (*Synarea*); therefore it was surprising that Clade III was not more distantly related to other *Porites*, but it illustrates how evolutionarily labile corallite morphology can be. Most molecular studies on corals have echoed similar findings that skeletal morphology is a poor indicator of underlying biodiversity. Morphology has been shown to be deceiving from species to family levels [e.g. [35,3]]. Fukami *et al*. [3] found unexpected morphological convergence at the family level that obscured deep divergence between the Atlantic and Pacific. Although divergence was deep between Atlantic and Pacific *Porites*, there was no clustering by oceanic region observed in this study, perhaps because the genus is ancient and was able to persist through regional mass extinction, and can survive conditions in the far Eastern Pacific, where few other corals exist.

It is difficult to determine if the highly evolutionarily flexible skeletal morphology observed in Clades I and II results from hybridization, incipient speciation, polymorphism, phenotypic plasticity, or a combination thereof. Discordance between the nuclear and mitochondrial data within Clade I (Figure 3) could be due to hybridization and nuclear introgression between several distinct maternal lineages, or recent speciation and contrasting rates of lineage sorting, or an artifact of differences in phylogenetic resolution among molecular markers. Hybridization or incipient speciation would be expected to result in less continuous trait variation than polymorphism or phenotypic plasticity. Vaughan [39] described 20 formae of *P. compressa *and 6 formae of *P. lobata*. *P. compressa *formae varied "continuously" from a semi-mounding form with a knobby surface to long slender branches, and *P. lobata *contained variation that was "enormous and bewildering". Although branching varieties in Clade I tend to occur more frequently in shallow water, both morphotypes frequently co-occur side-by-side in the same habitat. Further work is necessary to determine if these morphologically flexible clades represent hybrid or incipient species complexes, or if skeletal morphology is phenotypically plastic and/or a polymorphic trait maintained by natural selection in a heterogeneous environment.

This study represents important progress towards understanding the evolution and biodiversity of corals, and provides a foundation for future work. The genus *Porites *provides an excellent study system; it is abundant and ubiquitous in the tropics with an excellent fossil record, and corallite level characters that are well suited for quantitative morphometric analysis [40,41]. Future corallite-level morphometric work is likely to be highly informative about the nature of morphological variation observed in Clade I and II, and provide a means to test various alternative hypotheses and provide insights into coral evolution and biodiversity. The phylogenetic work is also an important starting point for studying the evolution of a variety of traits; for example, it is interesting to note that Clades I-V tend to contain broadcast spawning species such as *P. lobata, P. compressa*, and *P. cylindrica*, while Clades VI-XII tend to contain brooding species such as *P. astreoides*, *P. porites*, and *P. hawaiiensis*. This study also raises questions about the endemic status of several species, and demonstrates that patterns of biodiversity in this group are not as they appear and are in need of reevaluation. The range of *P. evermanni *may extend beyond Hawaii, because genetically indistinguishable and morphologically similar corals were found in Panama. Likewise, the Hawaiian endemics, *P. compressa *and *P. duerdeni*, were not genetically distinct and are morphologically very similar to more widespread branching species (*P. cylindrica *and *P. annae*). The putative new species *P. sp2*. is one of the most common varieties in American Samoa, but it was not previously known if it was genetically distinct. This study demonstrates that our understanding of coral biodiversity may be obscured by deceptive patterns of morphological variation.

## Conclusion

Untangling the coral 'species problem' will require a variety of tools and multiple lines of evidence. Here, we demonstrate that integration of taxonomy with analysis of multiple molecular markers reveals cryptic patterns of species diversity within the genus *Porites*. Cytochrome c oxidase subunit 1 (COI) has very low levels of polymorphism and has limitations as a useful tool for delimitating coral species [42]; however, it was informative when combined and compared in a multiple marker approach. The ITS region was particularly informative in this genus, and can be a valuable tool for elucidating patterns of evolution and biodiversity in corals. Since coral ecosystems are increasingly threatened, there is a need to characterize and understand coral species in terms of interbreeding groups as opposed to nominal morphological units. Our approach shows that morphological characters previously thought capable of delineating species must be reexamined to accurately understand patterns of evolution, endemism, and biodiversity in reef-building coral. Species definitions based solely on evolutionarily labile, polymorphic, or phenotypically plastic traits are likely to be misleading and confound attempts to identify, understand, and conserve coral biodiversity or to recognize its loss.

## Methods

Samples were collected by, or in consultation with individuals who are considered to be taxonomic authorities for a given geographic region. Voucher specimens were compared to museum type specimens and original species descriptions when possible. Sample identifications generally followed Veron [4]; however, since misidentification is a major problem in this genus, identifications should be considered tentative until the genus is revised, or until morphometric analysis (e.g. [40,41]) can verify similarity to type specimens. Voucher specimens and photographs are available upon request. Scanning electron microscope (SEM) images were obtained to examine selected samples of interest in further detail. Voucher collection, DNA extraction, PCR, cloning and sequencing methods were followed as described previously [21,23]. All samples were sequenced in both the forward and reverse directions. Several new primers were designed for this study with the aid of Primer 3 v 0.4.0 [43]. The primers amplify the cytochrome c oxidase subunit I mitochondrial gene (ZCO1 5'-TCA ACT AAT CAT AAA GAT ATT GGT ACG-3', ZCO1R 5'-TAA ACC TCT GGA TGC CCA AA-3') and the nuclear ribosomal ITS region (ITSZ1 5'-TAA AAG TCG TAA CAA GGT TTC CGT A-3', ITSZ2 5'-CCT CCG CTT ATT GAT ATG CTT AAA T-3'). The "putative mitochondrial control region" was amplified with primers from Volmer & Palumbi [6]. Sample locations, collector, and GenBank accession numbers are listed in Table 1; AY320289–AY320352, AY322575–AY322612, and AY458021–AY458063, were from previous work [23]. Two hundred and eighty four sequences were cloned and twenty-three sequences were direct sequenced from 91 individual coral colonies (there were few nucleotide differences between sequences that were cloned or direct sequenced from the same coral colony), averaging 3.4 sequences per individual. The mitochondrial markers had very low levels of polymorphism and did not amplify well in all samples, therefore only a few representative individuals were sequenced from each ITS group.

### Phylogenetic Analysis

The ITS sequences were aligned using the default parameters in MUSCLE v3.6 [44]. Gaps were coded as present or absent using the 'simple' gap coding method employed in GapCoder v1.0 [45]. Preliminary analysis (not shown) indicated that including coded gaps slightly increased bootstrap support for some deeper clades, with no major changes in topology. The complete ITS data set was run for 10 million generations in MrBayes 3.1.2 [46], until the standard deviation of the split frequencies was below 0.01, with a burn-in period of 2.5 million generations. Neighbor Joining (NJ), and Maximum Parsimony (MP) trees were generated in PAUP*4.01b.10 [47] with 1000 bootstrap replicates. NJ trees used the BioNJ method with the ML distance model selected in Modeltest V.3.7 [48]. ML models were selected by the Akaike information criterion for the nuclear (GTR+I+G) and mitochondrial (K81uf+G) partitions. In order to compare ITS and mitochondrial data sets, inclusive consensus sequences of all ITS molecular clones for 36 individuals that were successfully sequenced for both mitochondrial gene regions were generated in BioEdit v7.0.5.2 [49]. The three data partitions were concatenated, and compared using the incongruence length difference (ILD) test, implemented as partition homogeneity in PAUP*4.01b.10 [47]. The TBR branch swapping method was used with 1000 replications, random addition for 3 replicates, nchuck = 2, chuckscore = 1. Nuclear and mitochondrial data partitions were each run for 2 million generations in MrBayes 3.1.2 [46], with a burn-in of 5 hundred thousand generations. MEGA 4.0 [50] was used to estimate pair-wise genetic distance and to visualize phylogenetic trees.

## Authors' contributions

ZF designed and conceived of the study, performed the genetic analysis and drafted the manuscript. ZF and DB collected the genetic data, DB, CH, and RT helped draft the manuscript. All authors read and approved the final manuscript.
